# Supplementary figures and images for: Sargassum blooms in the Caribbean alter the trophic structure of the sea urchin Diadema antillarum
Source: PeerJ. 2019 Aug 30;7:e7589. doi: 10.7717/peerj.7589 (PMC6718159; doi:10.7717/peerj.7589)

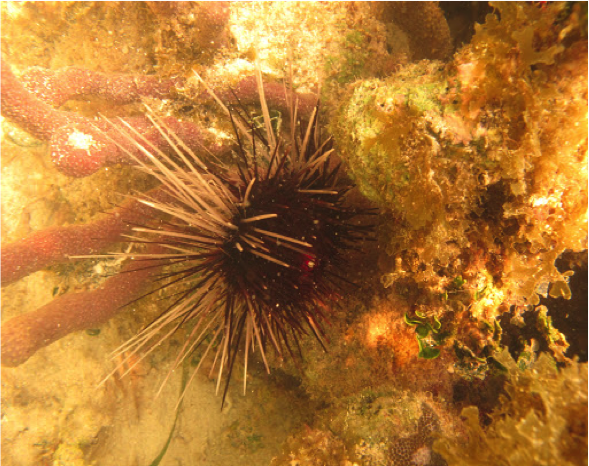

Supplement: Figure S1 [file peerj-07-7589-s001.png]

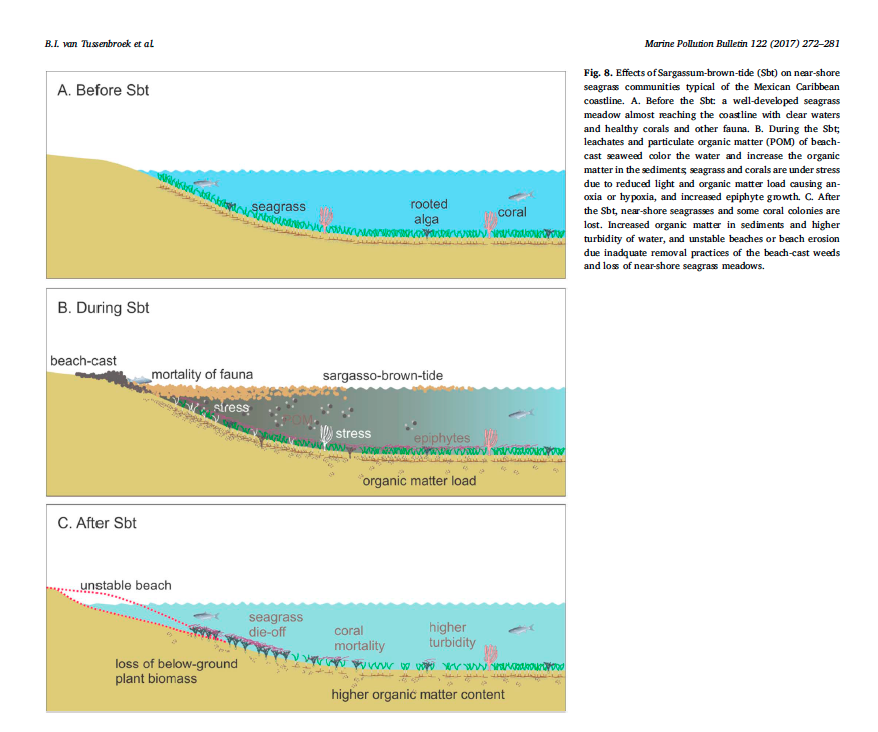

Supplement: File S1 — Confidential supplemental file. We based our Figure 2 on this Figure by van Tussenbroek et al., 2017. [file peerj-07-7589-s007.png]
