# Supplementary material for: Sargassum blooms in the Caribbean alter the trophic structure of the sea urchin Diadema antillarum
Source: PeerJ. 2019 Aug 30;7:e7589. doi: 10.7717/peerj.7589 (PMC6718159; doi:10.7717/peerj.7589)
Supplement: Table S1 [file peerj-07-7589-s006.docx]

**Supplemetal table 1.** Sampling collection of algae and sea urchins.

|  |  | **Mahahual** |  | **Xahuayxol** |  | **Xcalak** |  |
| --- | --- | --- | --- | --- | --- | --- | --- |
| **measurement** | **Sample type** | **WSE** | **USE** | **WSE** | **USE** | **WSE** | **USE** |
| Biomas (dry weight m-2)* | benthic macroalgae | x | x | x | x | x | x |
|  | local turf | x | x | x | x | x | x |
|  | *Sargassum fluitans* |  |  |  |  |  |  |
|  | *Sargassum natans* |  | x |  | x |  | x |
|  | *Sargassum*’s associated turf |  | x |  | x |  | x |
| Stable Istope Analysis (δ13C and δ15N) | benthic macroalgae | x | x | x | x | x | x |
|  | local turf | x | x | x | x | x | x |
|  | *Sargassum fluitans* | x | x | x | x | x | x |
|  | *Sargassum natans* |  |  |  |  |  |  |
|  | *Sargassum*’s associated turf | x | x | x | x | x | x |
| Dissolved Oxygen (mg l^-1^) | perpendicular transects from the coast to the back reef |  | x |  | x |  | x |

* nine quadrats (50 x 50 cm) per site.
