# Supplementary material for: Sargassum blooms in the Caribbean alter the trophic structure of the sea urchin Diadema antillarum
Source: PeerJ. 2019 Aug 30;7:e7589. doi: 10.7717/peerj.7589 (PMC6718159; doi:10.7717/peerj.7589)
Supplement: Supplemental Information 8 — Permission Letter written by Alejandro A. Aragón-Moreno [file peerj-07-7589-s008.pdf]

Baton Rouge, LA, United States of America, May 29 2019.

Title of PeerJ submission: ***Sargassum* blooms in the Caribbean alter the trophic structure of the sea urchin *Diadema antillarum***

Printed name of copyright holder: **Alejandro A. Aragón-Moreno**

Select copyrighted item: photographs | videos | other (please describe):

Map

I give my permission to PeerJ to publish my work, as described and/or appear below, under the CC-BY 4.0 license.

Signed,

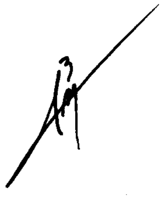

***Signature of copyright holder* Alejandro A. Aragón-Moreno**

Please supply the approved images(s) with the figure number(s) as they appear in the PeerJ submission OR A brief description of the video(s) including the title, description of content, length of video:

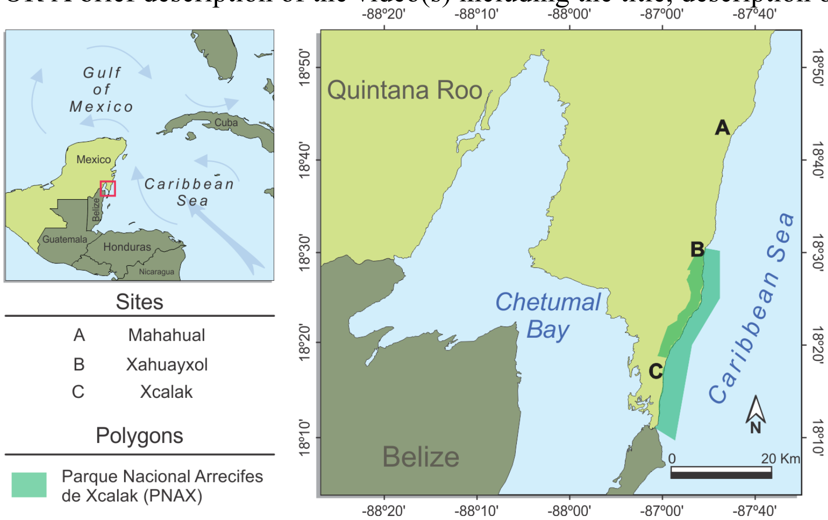

Figure 1. Study area and sampling localities at the south coast of Quintana Roo: Mahahual (A), Xahuayxol (B) and Xcalak (C). The green polygon represents the Marine Protected area Parque Nacional Arrecifes de Xcalak (PNAX).
